# Post-COVID-19 Syndrome: Nine Months after SARS-CoV-2 Infection in a Cohort of 354 Patients: Data from the First Wave of COVID-19 in Nord Franche-Comté Hospital, France

**DOI:** 10.3390/microorganisms9081719

**Published:** 2021-08-12

**Authors:** Souheil Zayet, Hajer Zahra, Pierre-Yves Royer, Can Tipirdamaz, Julien Mercier, Vincent Gendrin, Quentin Lepiller, Solène Marty-Quinternet, Molka Osman, Nabil Belfeki, Lynda Toko, Pauline Garnier, Alix Pierron, Julie Plantin, Louise Messin, Marc Villemain, Kevin Bouiller, Timothée Klopfenstein

**Affiliations:** 1 Infectious Disease Department, Nord Franche-Comté Hospital, 90400 Trevenans, France; pierre-yves.royer@hnfc.fr (P.-Y.R.); can.tipirdamaz@gmail.com (C.T.); ukaliq.eb@gmail.com (J.M.); vincent.gendrin@hnfc.fr (V.G.); lynda.toko@hnfc.fr (L.T.); alix.pierron@gmail.com (A.P.); louise.messin@orange.fr (L.M.); timothee.klopfenstein@hnfc.fr (T.K.); 2 Diabetology and Endocrinology Department, Nord Franche-Comté Hospital, 90400 Trevenans, France; hajer.zahra@gmail.com; 3 Virology Department, University Hospital of Besançon, 25000 Besançon, France; q1lepiller@chu-besancon.fr (Q.L.); solene.marty-quinternet@chu-besancon.fr (S.M.-Q.); 4 Faculty of Medicine of Tunis, University Tunis El Manar, Tunis 1007, Tunisia; molkaosman@gmail.com; 5 Internal Medicine Department, Groupe Hospitalier Sud Ile de France, 77000 Melun, France; belfeki.nabil@gmail.com; 6 Microbiology Department, Nord Franche-Comté Hospital, 90400 Trevenans, France; pauline.garnier@hnfc.fr (P.G.); julie.plantin@hnfc.fr (J.P.); marc.villemain@hnfc.fr (M.V.); 7 Infectious Disease Department, University Hospital of Besançon, 25000 Besançon, France; kbouiller@chu-besancon.fr

**Keywords:** post-COVID-19, long COVID-19, follow-up, fatigue, dyspnea, health care workers

## Abstract

(1) Background. Post-COVID-19 syndrome is defined as the persistence of symptoms after confirmed SARS-CoV-2 infection. (2) Methods. ANOSVID is an observational retrospective study in *Nord Franche-Comté* Hospital in France that included adult COVID-19 patients confirmed by RT-PCR from 1 March 2020 to 31 May 2020. The aim was to describe patients with post-COVID-19 syndrome with persistent symptoms (PS group) and to compare them with the patients without persistent symptoms (no-PS group). (3) Results. Of the 354 COVID-19 patients, 35.9% (*n* = 127) reported persistence of at least one symptom after a mean of 289.1 ± 24.5 days after symptom onset. Moreover, 115 patients reported a recurrence of symptoms after recovery, and only 12 patients reported continuous symptoms. The mean age of patients was 48.6 years (19–93) ± 19.4, and 81 patients (63.8%) were female. Patients in the PS group had a longer duration of symptoms of initial acute SARS-CoV-2 infection than patients in the no-PS group (respectively, 57.1 ± 82.1 days versus 29.7 ± 42.1 days, *p* < 0.001). A majority of patients (*n* = 104, 81.9%) reported three or more symptoms. The most prevalent persistent symptoms were loss of smell (74.0%, *n* = 94), fatigue (53.5%, *n* = 68), loss of taste (31.5%, *n* = 40), and dyspnea (30.7%, *n* = 39). These were followed by pain symptoms (26.8% (*n* = 34), 26.0% (*n* = 33), 24.4% (*n* = 31); headache, arthralgia, and myalgia, respectively). More than half of patients reporting persistent symptoms (58%, *n* = 73) were healthcare workers (HCWs). Among outpatients, this population was more present in the PS group than the no-PS group ((86.6%) *n* = 71/82 versus (72.2%) *n* = 109/151, *p* = 0.012). Post-COVID-19 syndrome was more frequent in patients with a past history of chronic rhinosinusitis (8.7% (*n* = 11%) versus 1.3% (*n* = 3), *p* < 0.001). No significant difference was found regarding clinical characteristics and outcome, laboratory, imaging findings, and treatment received in the two groups. (4) Conclusions. More than a third of our COVID-19 patients presented persistent symptoms after SARS-CoV-2 infection, particularly through loss of smell, loss of taste, fatigue, and dyspnea, with a high prevalence in HCWs among COVID-19 outpatients.

## 1. Introduction

One year after the coronavirus disease 2019 (COVID-19) outbreak, many questions remain, especially about the long-term clinical evolution after severe acute respiratory syndrome coronavirus 2 (SARS-CoV-2) infections. What is known so far is that patients with COVID-19 can sometimes report prolonged symptoms, even in young adults and children without underlying comorbidities and presenting initially mild-to-moderate forms of the disease [1,2]. Recently, there have been several reports of patients who reported no return to their previous state of health after having COVID-19 for several weeks or months, with variation in the duration of symptoms and clinical history [3]. Some authors referred to ‘long COVID-19 syndrome’ [2,4,5,6], ‘post-COVID-19 syndrome’ [7,8], or ‘post-acute COVID-19 syndrome’ [3,9], a syndrome that can possibly have an impact on the quality of life of patients [10]. The most common persistent symptoms in COVID-19 are fatigue and breathlessness [8,11,12]. Neurological symptoms, such as headaches [4,5,8,11,12]; otorhinolaryngological symptoms [11,13], such as olfactory and gustatory dysfunctions; psychiatric symptoms, such as concentration problems, sleep difficulties, and anxiety [11,14] are also common long-term consequences of COVID-19. Although considerable progress has been made in treating COVID-19, very little is known about the long-term consequences of this infection. For patients with post-COVID-19 syndrome, current medical therapies are essentially based on symptomatic treatment and rehabilitation [9]. Many scientific questions remain concerning the epidemiological, physiopathological, and therapeutic issues of this syndrome. The aim of our study was to describe persistence symptoms after SARS-CoV-2 infection.

## 2. Material and Methods

The ANOSVID study was sponsored by *Nord Franche-Comté* Hospital in France, and was designed in accordance with the declaration of Helsinki, and conducted in accordance with French legislation with approval obtained from the local ethics committee and the CPP (Comité de Protection des Personnes) SUD-EST IV, n° 20.10.08.63102.

ANOSVID was an observational retrospective study in *Nord Franche-Comté* Hospital in France. We included all adult inpatients and outpatients (≥18 years old) with a diagnosis of COVID-19 confirmed by RT-PCR on nasopharyngeal swabs from 1 March 2020 to 31 May 2020. RT-PCR was performed as previously described [15]. Patients’ consent was collected by phone calls. Patients who did not respond were called a second time, and a voice-message was left. In cases of a positive response, the patients filled in an online questionnaire. Data was collected during the first quarter of 2021. We excluded from this study any patient declining to participate in the study or expressing his or her opposition to data collection from hospital information systems, or those who did not respond or were unable to answer the online questionnaire. The aim of the ANOSVID study was to describe the clinical characteristics of COVID-19 patients with two main focuses: (i) anosmia and (ii) persistence of symptoms. Due to the large volume of data, the scientific committee suggested to focus firstly on the persistence of symptoms, which are analyzed in this work. The main outcome was to describe demographic characteristics, comorbidities, and the symptoms (clinical features and duration) of patients with a persistence of symptoms. The second outcome was to compare patients with a persistence of symptoms (PS group) and patients without a persistence of symptoms (no-PS group). The PS group was defined by patients who reported the persistence of at least one symptom (when they filled in the online questionnaire), and the no-PS group was defined by patients who fully recovered without any persistent symptoms.

Clinical data regarding demographic variables, comorbidities, COVID-19 characteristics, and persistence or no persistent symptoms at the date when the questionnaire was answered)) were collected through an online questionnaire sent by email (with a link to access it) and sent a second time in case of no reply. In case of hospitalization, hospitalization characteristics (duration of hospitalization, intensive care unit admission (ICU), outcome, and treatment) were collected through the medical record as biological, virological, and radiological findings. The persistence of symptoms was defined by the presence (at the date when the questionnaire was answered) of any symptoms related to SARS-CoV-2 infection, and which was not present before SARS-CoV-2 infection. In the PS group, patients who related a final date for symptoms due to SARS-CoV-2 infection before relapse were considered to have a “recurrence of symptoms” after an asymptomatic period. The patients who had a persistence of symptoms since the beginning of SARS-CoV-2 infection (without an asymptomatic period) were considered to have “continuous symptoms”.

Concerning the statistical analysis, continuous variables were expressed as mean and standard deviation (SD) and compared with Student’s *t*-test. Categorical variables were expressed as a number (%), and compared by a Chi2 test or Fisher’s exact test between the two groups (patients with persistent symptoms and patients without persistent symptoms). A *p*-value < 0.05 was considered significant. We used the SPSS v24.0 software (IBM, Armonk, NY, USA).

## 3. Results

During the study period, 354 COVID-19 patients were included in our facility. Of those patients, 227 (64.1%) were in the no-PS group, and 127 (35.9%) were in the PS group (Table 1). They had answered the questionnaire 289.1 (211–366) ± 24.5 days after symptoms onset in the mean.

### 3.1. Description of Patients with Persistent Symptoms of COVID-19

**Figure 1:**
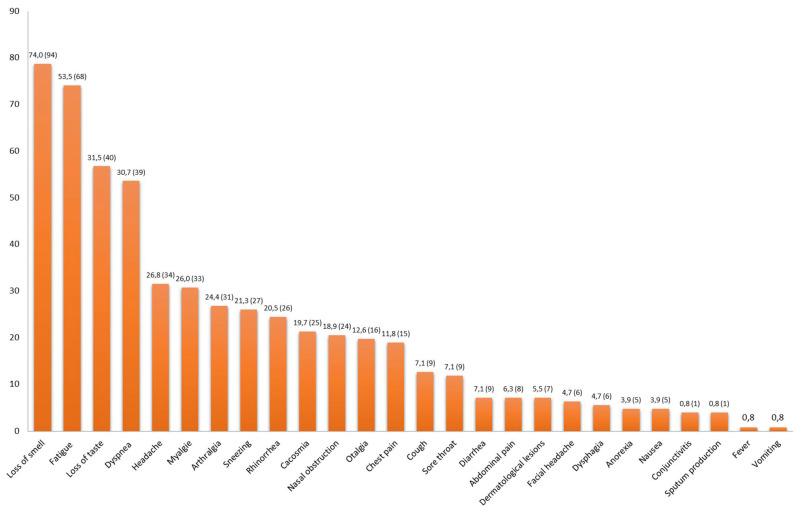
Proportion of persistent symptoms in the persistent symptom group (*n* = 127) after SARS-CoV-2 infection.

In the PS group (*n* = 127), the mean age of patients was 48.6 years (19–93) ± 19.4, and 81 (63.8%) of those patients were female. More than half of patients (57.5%, *n* = 73) were healthcare workers (HCWs). The mean body mass index (BMI) was 26.3 kg/m^2^ (16.6–47) ± 5.4. Furthermore, 78 patients (61.4%) had underlying comorbidities. The main comorbidities were otorhinolaryngological diseases (25.2%, *n* = 32), cardiovascular diseases (22%, *n* = 28), and respiratory diseases (21.3%, *n* = 27). Moreover, eight patients (6.3%) had a past history of depressive disorders. Only a third of patients were hospitalized (35.4%, *n* = 45) with a mean duration of 11.5 days (1–45) ± 10.5. Of those patients, five (11.1%) were transferred to the ICU and mechanically ventilated. Among the 127 patients, 115 reported a recurrence of symptoms after recovery, and only 12 patients reported continuous symptoms (persistence of symptoms since the beginning of SARS-CoV-2 infection without an asymptomatic period). The most common persistent symptoms were loss of smell (74.0%, *n* = 94), fatigue (53.5%, *n* = 68), loss of taste (31.5%, *n* = 40), and dyspnea (30.7%, *n* = 39) followed by pain symptoms (26.8% (*n* = 34), 26.0% (*n* = 33), 24.4.8% (*n* = 31); headache, arthralgia and myalgia respectively). Concerning loss of smell, 36/121 COVID-19 inpatients (29.8%) versus 64/233 outpatients (27.5%) presented loss of smell as a persistent symptom, indicating no significant difference (*p* = 0.369). Gastro-intestinal symptoms and fever were present in <10% and <1% of cases, respectively (Figure 1). More than one-third of patients (35%, *n* = 44) had at least five symptoms with a longer mean duration of 90.3 days (9–335) ± 103.0 (Table 2).

### 3.2. Comparison of Two Groups (Table 2)

The mean age was 48.6 years ±19.4 in PS group and 50.1 years ±18.4 in no-PS group (*p* = 0.47), without sex predominance. We only found a significant difference in patients between 61 and 70 years (15.7% of patients in the PS group (*n* = 20) versus 8.8% in the no-PS group (*n* = 20), *p* = 0.048). No significant difference was found regarding BMI, current smoking, or comorbidities among the two groups, except for chronic rhinosinusitis, which was more often found in the PS group than the no-PS group (8.7% (*n* = 11%) versus 1.3% (*n* = 3), *p* < 0.001).

Patients in the PS group had a longer duration of symptoms of initial acute SARS-CoV-2 infection than patients in the no-PS group (respectively, 57.1 ± 82.1 days versus 29.7 ± 42.1 days, *p* < 0.001). There were no other significant differences between the two groups concerning clinical characteristics, biological findings, imaging findings, and outcome.

Among the 233 outpatients, HCWs were more presented in the PS group than in the no-PS group ((86.6%) *n* = 71/82 versus (72.2%) *n* = 109/151, *p* = 0.012).

## 4. Discussion

Post-COVID-19 syndrome is currently defined as the presence of symptoms for more than 12 weeks developed during or after SARS-CoV-2 infection and which are not explained by an alternative diagnosis [7]. Thus, for all patients presenting with prolonged symptoms following COVID-19, other causes of acute decompensation of underlying chronic conditions should be ruled out first.

Among the 354 patients included in our cohort study, 36% of patients reported the presence of one or more symptoms after at least 9 months (in the mean) after COVID-19 onset. The most common symptoms were fatigue, dyspnea, and pain symptoms, which is consistent with the literature thus far [1,2,3,4,11,12,16]. These findings suggested systemic chronic inflammation with abnormal pro-inflammatory cytokine expression, which was observed in patients following COVID-19 [17]. Chronic fatigue syndrome involved multifactorial pathomechanisms. One hypothesis is that fatigue is due to alterations in mitochondrial structure, metabolism, and energy production within muscle tissues [17]. Further research in this area could provide new insights into the understanding of chronic fatigue syndrome. However, dyspnea in the context of post-COVID-19 syndrome is recognized as having a strong orthostatic component [17], or as being secondary to post-viral injury in some cases [18,19]. Patients in the PS group had a longer duration of symptoms of acute SARS-CoV-2 infection than patients in the no-PS group (57 days versus 30 days, *p* < 0.001). It is possible that patients with a longer SARS-CoV-2 infection are more susceptible to having persistent symptoms. It would be interesting to have a virological follow-up with RT-PCR SARS-CoV-2 in these patients to emphasize this hypothesis.

Our populations did not differ in sex, age (except for the age range for the group between 61 and 70 years), or demographic characteristics. Some authors suggested that the persistence of symptoms could be explained by the delay of SARS-CoV-2 RNA clearance in older patients. This hypothesis cannot be confirmed in our study in the absence of follow-up RT-PCR screening of our patients. Moreover, there was no correlation between initial Ct values of viral load and post-COVID-19 syndrome in our study. We have two assumptions that may explain why this age group (particularly between 61 and 70 years) seems to express the persistence of symptoms. Firstly, some authors thought that post-COVID-19 syndrome may affect patients at any age, but it seems to be more common in elderly patients with a higher risk of sarcopenia, malnutrition, depression, and delirium [9]. Secondly, the presence of persistent symptoms could possibly be helpful in anticipating an early departure from the workforce, especially as France is beginning to recognize persistent symptoms after COVID-19 as an occupational disease [20]. Furthermore, some authors suggest that post-COVID-19 syndrome is more frequent in outpatients than in inpatients. We have not found this difference in our study; the small sample size could explain this finding.

Among outpatients, HCWs were significantly more present in the group of patients with post-COVID-19 syndrome, which is consistent with the medical literature [8,21]. On the other hand, the high number of HCWs with mild-to-moderate symptoms in our cohort could be explained by the young age of patients with no underlying comorbidities. The hypothesis that prolonged and repeated exposure to SARS-CoV-2 contributes to a higher viral load in this population has already been discussed in the literature [22], but this theory seems unlikely as exposure to infection leads to immunity in most cases. On the other hand, post-traumatic stress disorder, peritraumatic dissociation, and anxiety disorders may occur more frequently in HCWs due to the caring responsibilities in this epidemic context [9,23].

Riestra-Ayora et al. concluded that one of the most persistent symptoms in COVID-19 patients, especially in HCWs, are olfactory and gustatory dysfunctions [13]. It has already been shown that both anosmia and dysgeusia are more frequently described in the European population than in the Asian population [24,25]. In this cohort, these otorhinolaryngological symptoms were some of the most common persistent symptoms after a 9-month follow-up. This is likely a result of prolonged primary chemosensory dysfunction. This can also possibly explain the high prevalence of persistent symptoms in patients with chronic rhinosinusitis in this population. We do not report any significant difference in hospitalization rate and duration, ICU admission, and MV. Only 11% of ICU patients reported persistent symptoms in our cohort. However, it is known that patients presenting with critical illness and acute respiratory distress syndrome related to COVID-19 can develop post-intensive care syndrome afterwards. This syndrome may combine mental disorders, cognitive impairment, memory loss, muscle weakness, dysphagia, and reduced quality of life [26,27].

It is important for all patients presenting with prolonged symptoms following COVID-19 that other causes of acute decompensation of underlying chronic conditions be ruled out first.

Limitations included possible undetected pre-COVID-19 abnormalities in our patients, and the exclusion of some patients with severe comorbidities who were not able to respond to our questionnaire. Another limitation was the inclusion of COVID-19 patients during the first wave of the epidemic in France. Currently, we do not have any data about the occurrence of the new United Kingdom (20I/501Y.V1) and South African (20H/501Y.V2) SARS-CoV-2 variants and post-COVID-19 syndrome. This can also explain why among our inpatients, treatment was principally based on antibiotics and hydroxychloroquine, while steroids were underused. Furthermore, the high percentage of outpatients is biased due to the high number of HCWs. Our follow-up was only based on a questionnaire, which is an issue of subjectivity. However, investigations such as lung functions tests and chest CT scan findings were normal in most cases of patients with post COVID-19 symptoms in the medical literature [1]. Finally, it is necessary to remember that the upper respiratory tract is the gateway for many different pathogenic viruses such as SARS-CoV-2, but it is also home to commensal and potentially pathogenic bacterial communities. In this context, viral, viral/bacterial co-infections, or superinfections may explain the persistence of symptoms in some patients [28,29].

## 5. Conclusions

This study confirms the high prevalence of persistent symptoms in HCWs among COVID-19 outpatients. A longer duration of initial symptoms after COVID-19 infection could also explain the prevalence of the post-COVID-19 syndrome.

## Figures and Tables

**Table 1:** Demographic, clinical characteristics, laboratory, and imaging findings in 354 COVID-19 patients with or without persistent symptoms after SARS-CoV-2 infection, *Nord Franche-Comte Hospital, France*.

			No-PS Group (*n* = 227)	PS Group (*n* = 127)	Total (*n* = 354)	*p*-Value
			(64.1%)	(35.9%)	(100%)	
Demographic and baseline characteristics						
Age, years (mean, extremes, SD)			50.1 (19–98) ± 18.4	48.6 (19–93) ± 19.4	49.6 (19–98) ± 18.7	0.472
		(18–30)	41 (18.1)	30 (23.6)	71 (20.1)	0.210
		(31–40)	32 (14.1)	18 (14.2)	50 (14.1)	0.984
		(41–50)	50 (22)	23 (18.1)	73 (20.6)	0.382
		(51–60)	46 (20.3)	19 (15)	65 (18.4)	0.216
		**(61–70)**	**20 (8.8)**	**20 (15.7)**	**40 (11.3)**	**0.048**
		(71–80)	22 (9.7)	8 (6.3)	30 (8.5)	0.272
		(81–90)	12 (5.3)	8 (6.3)	20 (5.6)	0.692
		>90	4 (1.8)	1 (0.8)	5 (1.4)	0.411
Sex (Number, %)		Male	85 (37.4)	46 (36.2)	131 (37.0)	0.819
		Female	142 (62.6)	81 (63.8)	223 (63.0)	0.819
HCWs (Number, %)		Total	112 (49.3)	73 (57.5)	185 (52.3)	0.141
		Inpatients	3/76 (3.9)	2/45 (4.4)	5/121 (4.1)	0.617
		**Outpatients**	**109/151 (72.2)**	**71/82 (86.6)**	**180/233 (77.3)**	**0.012**
BMI (kg/m^2^)		Total (mean, extremes, SD)	26.7 (15.9–43.2) ± 5.3	26.3 (16.6–47) ± 5.4	26.5. (15.9–47) ± 5.3	0.472
		<18.5	6 (2.7)	6 (4.7)	12(3.4)	0.234
		(18.5–25)	92 (40.9)	52 (40.9)	144 (40.9)	0.992
		(25–30)	74 (32.9)	40 (31.5)	114 (32.4)	0.780
		>30	53 (23.6)	29 (22.8)	82 (23.3)	0.878
Pregnancy (Number, %)			1 (0.4)	3 (2.4)	4 (1.1)	0.101
Current smoking (Number, %)			29 (12.8)	12 (9.4)	41 (11.6)	0.348
Comorbidities (Number, %)		No	93 (41)	49 (38.6)	142 (40.1)	0.660
Cardio-vascular diseases (Number, %)		Total	71 (31.3)	28 (22)	99 (28)	0.060
		Arterial hypertension	48 (21.1)	21 (16.5)	69 (19.5)	0.294
		Heart failure	12 (5.3)	2 (1.6)	14 (4)	0.086
		Others ^1^	18 (7.9)	7 (5.5)	25 (7.1)	0.394
Diabetes mellitus			18 (7.9)	6 (4.7)	24 (6.8)	0.250
Chronic kidney failure (Number, %)			6 (2.6)	3 (2.4)	9 (2.9)	0.872
Neurologic diseases (Number, %)			15 (6.6)	6 (4.7)	21 (5.9)	0.472
Psychiatric disorders (Number, %)		Depressive disorders	10 (4.4)	8 (6.3)	18 (5.1)	0.437
		Others ^2^	2 (0.9)	0 (0)	2 (0.6)	0.411
Malignancy (Number, %)		Past history of malignancy	20 (8.8)	10 (7.9)	30 (8.5)	0.762
		Treated actually	2 (0.9)	2 (1.6)	4 (1.1)	0.453
Respiratory diseases (Number, %)		Total	38 (16.5)	27 (21.3)	66 (18.6)	0.345
		COPD	7 (3.1)	2 (1.6)	9 (2.5)	0.314
		Asthma	26 (11.5)	19 (15)	45 (12.7)	0.342
		Others ^3^	5 (2.2)	5 (3.9)	10 (2.8)	0.266
ENT diseases (Number, %)		Total	48 (21.1)	32 (25.2)	80 (22.6)	0.382
		Rhinosinusitis nasal polyps	2 (0.9)	3 (2.4)	5 (1.4)	0.248
		Surgical rhinoplasty	5 (2.2)	4 (3.1)	9 (2.9)	0.413
		Allergic rhinitis	40 (17.6)	19 (15)	59 (16.7)	0.519
		**Chronic rhinosinusitis**	**3 (1.3)**	**11 (8.7)**	**14 (4)**	**<0.001**
Clinical characteristics and outcome, laboratory and imaging findings						
**Duration of symptoms of SARS-CoV-2 infection (days) (mean, extremes, SD)**			**29.7 (1–283) ± 42.1**	**57.1 (0–335) ± 82.1**	**39.1 (0–335) ± 60.2**	**<0.001**
Crackling Sounds heard on pulmonary auscultation (Number, %)			56/76 (73.7)	33/45 (73.3)	89/121 (73.6)	0.966
Hospitalization (Number, %)			76 (33.5)	45 (35.4)	121 (34.2)	0.710
Duration of hospitalization (days) (mean, extremes, SD)			14.2 (1–128) ± 20.6	11.5 (1–45) ± 10.5	13.2 (1–128) ± 17.6	0.415
Transferred to ICU (Number, %)			13/76 (17.1)	5/45 (11.1)	18/121 (14.9)	0.371
Mechanical ventilation (Number, %)			13/76 (17.1)	5/45 (11.1)	18/121 14.9)	0.371
Laboratory data on admission (mean, extremes, DS) in hospitalized patients		White-cell count/mm^3^ (4000–10,000/mm^3^)	7795 (2660–23,950) ± 3.4	7161 (2790–13,340) ± 2.5	7561 (2660–23,950) ± 3.5	0.344
		Lymphocytes/mm^3^ (1500–4000/mm^3^)	0.889 (0.210–2.380) ± 0.4	0.950 (0.150–2.700) ± 0.5	0.911 (0.150–2700) ± 0.5	0.485
		Hemoglobin, g/dL (13.5–17.5 g/dL)	13.9 (10.9–18.2) ± 1.5	13.5 (10.4–19.1) ± 1.8	13.8 (10.4–19.1) ± 1.6	0.208
		Creatinine, μmol/L (65–120 μmol/L)	83.2 (46–403) ± 48.5	77.2 (43–139) ± 20.9	81.0 (43–403) ± 40.5	0.434
		Alanine aminotransferase, U/L (8–45 U/L)	53.0 (12–230) ± 43.7	53.5 (12–175) ± 46.6	53.2 (12–230) ± 44.5	0.964
		Aspartate aminotransferase, U/L (10–40 U/L)	56.5 (11–292) ± 45.0	53.9 (11–193) ± 41.5	55.6 (11–292) ± 43.6	0.767
		D-dimer, ng/mL (<500 ng/mL)	1695 (<500–34905)	2169 (<500–58190)	1871 (<500–58190]	0.719
		C-reactive protein (CRP), mg/L	121.1 (1–478) ± 94.2	117.2 (1–490) ± 96.6	119.6 (1–490) ± 94.7	0.828
		CRP >100mg/L (Number, %)	37/76 (48.7)	23/45 (51.1)	60/121 (49.6)	0.769
RT-PCR SARS-CoV-2 CT (mean, extremes, SD)			27.0 (16.4–37.6) ± 5.4	27.2 (17.3–34.2) ± 5.3	27.1 (16.4–37.6) ± 5.3	0.901
Radiologic data	Thoracic imaging features	GGO	58/60 (96.7)	30/32 (93.8)	88/92 (95.7)	0.434
		Consolidation opacities	45/60 (75)	22/32 (68.8)	67/92 (72.8)	0.521
		Crazy-paving sign	28/60 (46.7)	13/32 (40.6)	41/92 (44.6)	0.579
		Extension < 25%	30/60 (50.0)	14/32 (43.8)	44/92 (47.8)	0.568
		Extension >50%	8/60 (13.3)	3/32 (9.4)	11/92 (12.0)	0.424
Treatment received						
Antibiotics *			64/76 (84.2)	35/45 (77.8)	99/121 (81.8)	0.375
Hydroxychloroquine			51/76 (67.1)	26/45 (57.8)	77/121 (63.6)	0.303
Lopinavir/Ritonavir			5/76 (6.6)	2/45 (4.4)	7/121 (5.8)	0.480
Steroids (Dexamethasone)			11/76 (14.5)	6/45 (13.3)	17/121 (14.0)	0.862
Anti-IL-6 (Tocilizumab)			3/76 (3.9)	3/45 (6.7)	6/121 (5.0)	0.396

Bold: significant difference (*p* < 0.05), Abbreviations (alphabetic order): Anti-IL-6: anti-interleukine-6 receptor; BMI: body mass index; COPD: chronic obstructive pulmonary disease; CT: cycle threshold; CRP: C-reactive protein; D: days; ENT: Ear, Nose and Throat; HCWs: Health care workers; ICU: intensive care unit; GGO: Ground-glass opacity; NA: not available; PS: persistent symptoms; RT-PCR: reverse transcription Polymerase Chain Reaction; SARS-CoV-2: severe acute respiratory syndrome coronavirus 2; SD: standard derivation; ^1^ Defined by: cardiac arrhythmia, coronary heart disease, peripheral arterial obstructive disease and thromboembolic disease ^2^ Defined by: bipolar, anxiety and panic disorders ^3^ Defined by: community acquired pneumonia, emphysema and obstructive sleep apneas. * Concerning antibiotics, we only use β-lactams antimicrobials (amoxicillin/clavulanate 3 g per day (IV or orally) or IV ceftriaxone 1 g per day. Fluoroquinolones (levofloxacin 500 mg per day (IV or orally were only used in case of β-lactams allergy.

**Table 2:** COVID-19 patients with persistent symptoms after SARS-CoV-2 infection, *Nord Franche-Comte Hospital, France*.

Persistent Symptoms (Number)	Number of Patients with Persistent Symptoms (%)	Duration of SARS-CoV-2 Acute Infection (Mean, Extremes, SD), Days
1	7 (5.5)	13.5 (7–19) ± 5
2	16 (12.6)	59.9 (8–279) ± 86.3
3	36 (28.3)	55.5 (7–303) ± 85.4
4	24 (18.9)	37.4 (10–119) ± 25.8
>5	44 (34.6)	90.3 (9–335) ± 103.0
Total	127 (100)	43.8 (7–335) ± 62.2

## Data Availability

Data available on request due privacy to restrictions. The data presented in this case study are available on request from the corresponding author.
